# Anodic Fabrication of Ti-Ni-O Nanotube Arrays on Shape Memory Alloy

**DOI:** 10.3390/ma7043262

**Published:** 2014-04-22

**Authors:** Qiang Liu, Dongyan Ding, Congqin Ning

**Affiliations:** 1 Institute of Microelectronic Materials and Technology, School of Materials Science and Engineering, Shanghai Jiao Tong University, Shanghai 200240, China; E-Mail: lqshjt@sjtu.edu.cn; 2 State Key Laboratory of High Performance Ceramics and Superfine Microstructure, Shanghai Institute of Ceramics, Chinese Academy of Sciences, Shanghai 200050, China; E-Mail: cqning@mail.sic.ac.cn

**Keywords:** TiNi alloy, anodization, nanotubes, thermal stability, Raman

## Abstract

Surface modification with oxide nanostructures is one of the efficient ways to improve physical or biomedical properties of shape memory alloys. This work reports a fabrication of highly ordered Ti-Ni-O nanotube arrays on Ti-Ni alloy substrates through pulse anodization in glycerol-based electrolytes. The effects of anodization parameters and the annealing process on the microstructures and surface morphology of Ti-Ni-O were studied using scanning electron microscope and Raman spectroscopy. The electrolyte type greatly affected the formation of nanotube arrays. A formation of anatase phase was found with the Ti-Ni-O nanotube arrays annealed at 450 °C. The oxide nanotubes could be crystallized to rutile phase after annealing treatment at 650 °C. The Ti-Ni-O nanotube arrays demonstrated an excellent thermal stability by keeping their nanotubular structures up to 650 °C.

## 1. Introduction

TiO_2_ is one of the main semiconducting materials used for a variety of applications including photocatalysts, gas sensors, water purification and solar energy applications [1–4]. However, TiO_2_ is well-known as an n-type semiconductor with a wide band gap. It can only absorb in the UV regime and thus significantly limits its widespread application [5,6]. In order to improve the electrical and structural properties of TiO_2_ and thus broaden its application field, various kinds of decoration techniques have been carried out. Among the diverse approaches, doping of the oxide is one of the most efficient ways to obtain improved physical and chemical properties. For example, many metal elements (Fe, Cu, Cr, *etc*.) [7–9], and nonmetal elements (B, C, N, *etc*.) [10–12], have been used as dopants.

One-dimensional nanostructured materials, especially nanotubes, offer great potential for various applications such as sensing, water photolysis, solar energy conversion and electrochemical energy storage [13]. The anodization process has shown its excellent capability in fabricating various kinds of nanostructures. Allam *et al*. [14] reported a unique route for fabricating large-scale vertically oriented TiO_2_ nanotube arrays through one-step anodization of alloy substrates. Mor *et al*. [15] fabricated Ti-Fe-O nanotube arrays on Ti-Fe films. Liu *et al*. [16] fabricated highly ordered Ti-Nb-O nanotubes on Ti35Nb alloy. Allam *et al*. [17] also fabricated Ti-Nb-Zr-O nanotubes for enhanced hydrogen generation by water photoelectrolysis.

Unlike the anodization of other Ti alloys, anodization of Ti-Ni films in traditional electrolytes usually could not yield nanotube arrays because Ti-Ni-O could easily dissolve in aqueous solutions. To date, few works have been reported on anodic fabrication of Ti-Ni-O nanotube arrays on Ti-Ni shape memory alloy substrates. In this work, highly ordered Ti-Ni-O nanotube arrays were successfully grown on Ti-Ni alloy substrates through pulse anodization in glycerol-based electrolytes. The influence of anodization voltages on the microstructures and thermal stability of Ti-Ni-O nanotube arrays was investigated.

## 2. Results and Discussion

### 2.1. Fabrication of Ti-Ni-O Nanotube Arrays

**Figure 1.**
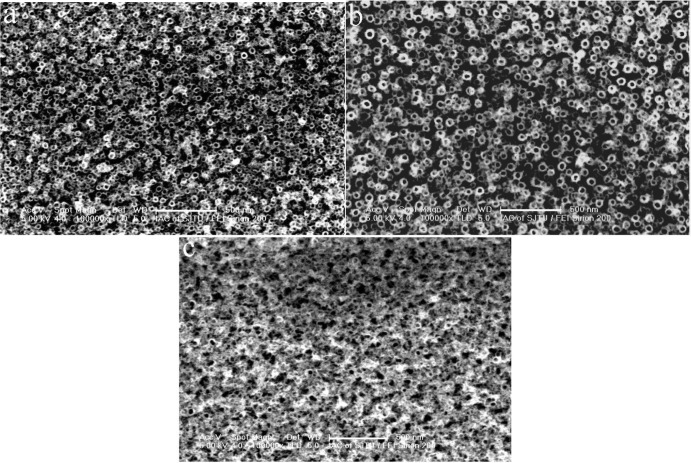
Top view images of as-anodized Ti-Ni-O nanostructures fabricated at different pulse voltages in a non-aqueous electrolyte of 5% ethylene glycol/glycerol containing 0.30 M (NH_4_)_2_SO_4_ and 0.4 M NH_4_F, (**a**) 30V; (**b**) 40 V; (**c**) 50 V.

Figure 1 shows top view images of the anodic Ti-Ni-O nanostructures fabricated at different pulse voltages in the non-aqueous electrolyte of 5% ethylene glycol/glycerol containing 0.30 M (NH_4_)_2_SO_4_ and 0.4 M NH_4_F. When the anodization voltage was 30 V, the surface of the Ti-Ni-O presented an irregular nanoporous structure. When the anodization voltage was increased to 40 V, the nanotubes with a small diameter formed. Some regions could not have complete nanotubes because of corrosion of the nanotubes. When the anodization voltage was 50 V, the nanotubes were completely corroded.

**Figure 2.**
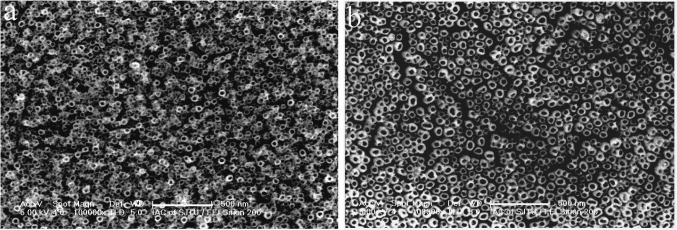

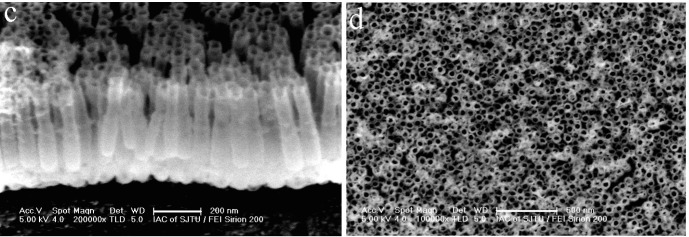
Top view and cross section images of as-anodized Ti-Ni-O nanostructures fabricated at different pulse voltages in a non-aqueous electrolyte of 5% ethylene glycol/glycerol containing 0.30 M (NH_4_)_2_SO_4_ and 0.2 M NH_4_F, (**a**) 30 V; (**b**,**c**) 40 V; (**d**) 50 V.

In order to investigate the effect of fluoride concentration on the formation of nanotube arrays, non-aqueous electrolyte containing lower fluoride species was also evaluated. Figure 2 shows the top view and cross section images of as-anodized oxide nanostructures fabricated at different pulse voltages in the non-aqueous electrolyte of 5% ethylene glycol/glycerol containing 0.30 M (NH_4_)_2_SO_4_ and 0.2 M NH_4_F. When the Ti-Ni substrate was anodized at 30 V, the top surface of the oxide layer presented uniform nanoporous structures. When the anodization voltage was increased to 40 V, highly ordered Ti-Ni-O nanotube arrays could form. As shown in Figure 2b,c, the average inner diameter and length of the nanotubes were 55 and 450 nm, respectively. When the Ti-Ni foil was anodized at 50 V, the top surface of the Ti-Ni-O nanotube arrays appeared partially damaged because of a rapid chemical dissolution reaction. However, self-organized nanotube arrays were still apparent.

### 2.2. Thermal Stability of Ti-Ni-O Nanotube Arrays

**Figure 3.**
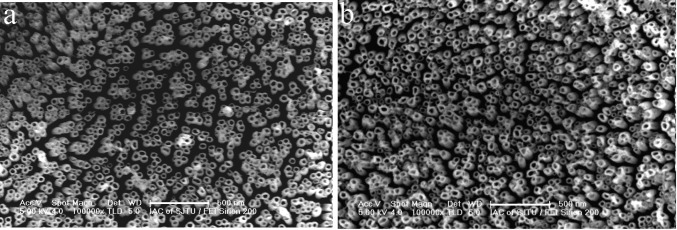

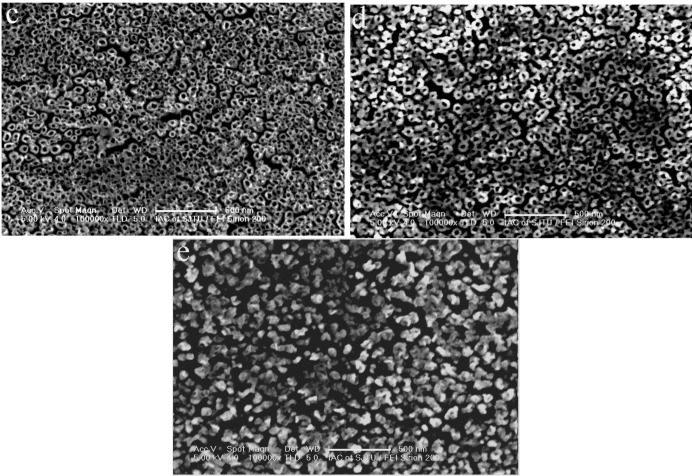
Top view images of Ti-Ni-O films annealed at different temperatures (**a**) 500 °C; (**b**) 550 °C; (**c**) 650 °C; (**d**) 700 °C; (**e**) 800 °C

Figure 3 shows top view images of the Ti-Ni-O nanofilms fabricated at 40V in the non-aqueous electrolyte of 5% ethylene glycol/glycerol containing 0.30 M (NH_4_)_2_SO_4_ and 0.2 M NH_4_F and further annealed at different temperatures. In comparison with the anodic Ti-Ni-O nanotube arrays, a heat treatment at 500 °C in air did not change the surface morphology of the Ti-Ni-O nanotube arrays (Figure 3a). With increase of the heat treatment temperature to 550 °C, the Ti-Ni-O nanotubes could still keep their highly ordered nanotubular structures (Figure 3b). When the heat treatment temperature increased to 650 °C, only slight change of the Ti-Ni-O nanotubes was found. As shown in Figure 3c, the top ends of some nanotubes collapsed, although nanotubular structures could be still observed. This suggests that the Ti-Ni-O nanotube arrays could bear such a temperature. When the heat-treatment temperature increased to 700 °C, the nanotubular structure was destroyed and some nanotubes totally collapsed (Figure 3d). With increase of the heat-treatment temperature to 800 °C, the nanotubular structure totally collapsed without showing any trace of nanotubes (Figure 3e). As for pure TiO_2_ nanotubes, the nanotubular structure was found to be totally destroyed when the heat temperatures was 650 °C in air [18]. Obviously, Ni-doping could enhance the thermal stability of in TiO_2_ nanotubes.

### 2.3. Structural Properties

**Figure 4.**
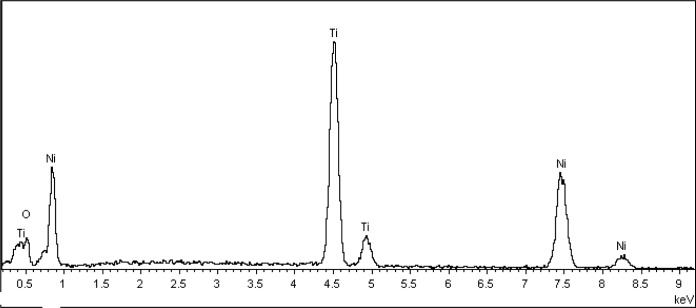
EDX pattern of the as-annealed Ti-Ni-O nanotubes grown on Ti-Ni alloy substrates.

Figure 4 shows EDX pattern of the as-annealed Ti-Ni-O nanotubes grown on Ti-Ni alloy substrates. The Ti-Ni-O was mainly composed of Ti, Ni and O elements. As shown in Table 1, atomic percentages of the Ti, Ni and O elements were 26.26%, 6.83% and 66.87%, respectively. The atomic percentages of Ni and Ti elements in the as-annealed Ti-Ni-O films were much lower than those in the original Ti-Ni alloy substrate. Furthermore, the Ni content was also much lower than the Ti content in the as-annealed Ti-Ni-O films. This phenomenon could be explained by considering the difference in the dissolution rate for different kinds of oxides during the anodization process. Ni oxide could be easily dissoluted in the electrolyte solution [19,20].

**Figure 5.**
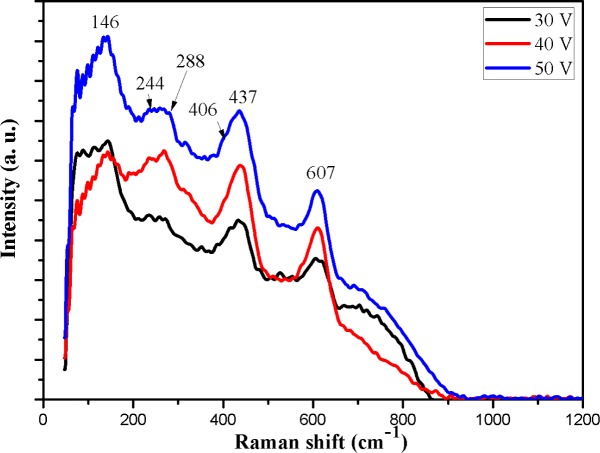
Raman shift of Ti-Ni-O fabricated at different pulse voltages, (**a**) 30 V; (**b**) 40 V; (**c**) 50 V.

Structural properties of the Ti-Ni-O nanofilms were analyzed using Raman spectroscopy. Figure 5 shows Raman spectra of the Ti-Ni-O nanofilms fabricated at different pulse voltages. All of the as-anodized Ti-Ni-O samples had been annealed at 500 °C for one hour in air. According to literatures [21,22], six Raman active modes for undoped anatase TiO_2_ could be found at 144 (E_g_), 197 (E_g_), 399 (B_1g_), 513 (A_1g_), 519 (B_1g_) and 639 (E_g_) cm^−1^. Typical Raman peaks of rutile TiO_2_ were detected at 143 (B_1g_), 235(E_g_), 447(E_g_) and 612 (A_1g_) cm^−1^.

For the oxide nanotubes fabricated here, the Raman bands of the Ti-Ni-O did not appear at the same frequencies as those of the undoped TiO_2_ because the Ni-doping had changed the vibrational modes of TiO_2_. For the as-annealed Ti-Ni-O nanotubes, the Raman peaks around 146, 244, 288, 406, 437 and 607 cm^−1^ could be detected (Figure 5). The peak at 146 cm^−1^ and a small shoulder around 406 cm^−1^ corresponded to the anatase phase. The peaks found at 244, 437 and 607 cm^−1^ corresponded to rutile phase. The peak at 288 cm^−1^ should be attributed to titanate bands [23,24]. Obviously, both the anatase and rutile phases could coexist in the Ti-Ni-O nanotube layer after the heat-treatment at 500 °C.

**Figure 6.**
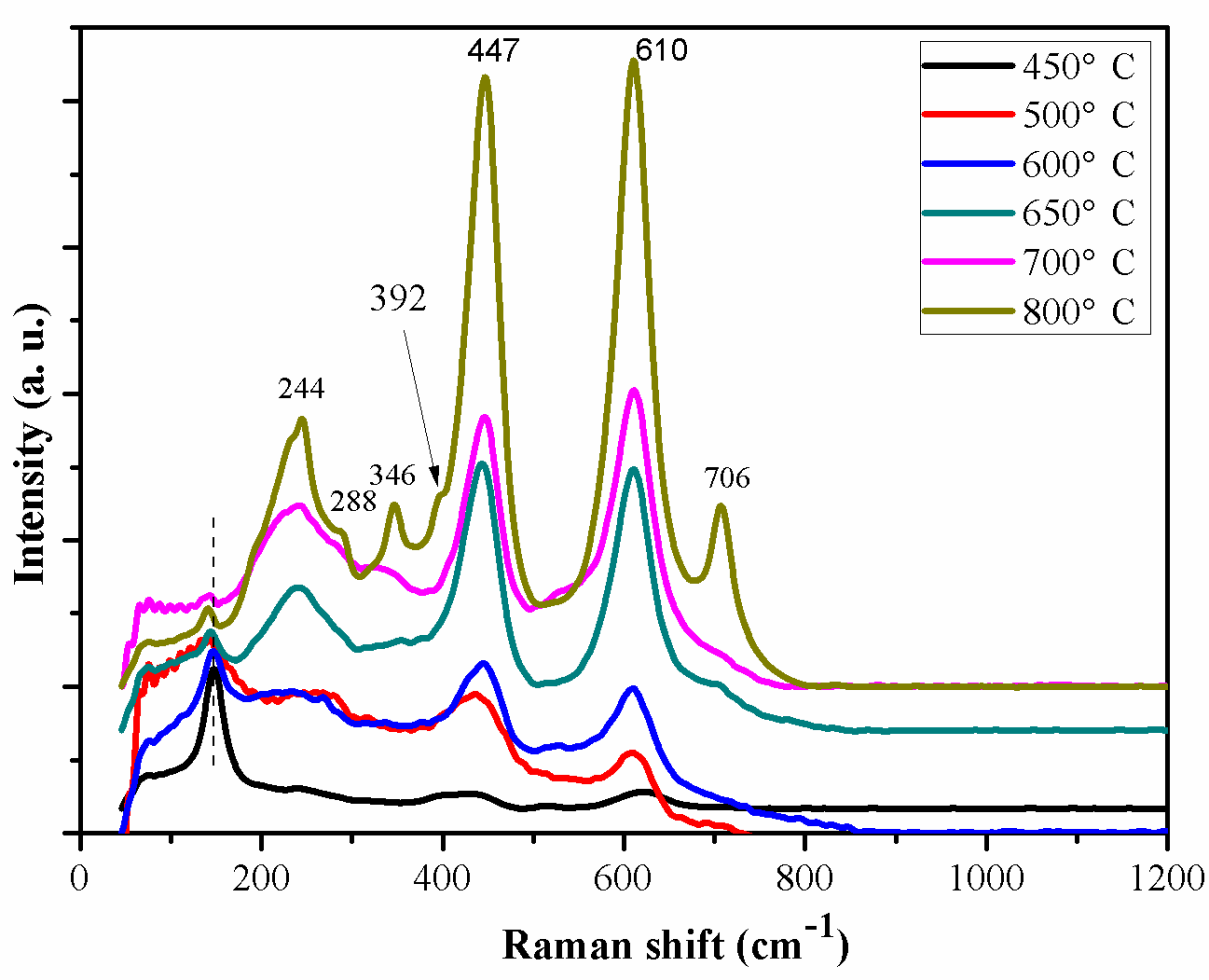
Raman shift of Ti-Ni-O samples annealed at different temperatures.

Figure 6 presents Raman spectra of the as-anodized Ti-Ni-O nanofilms annealed at 450, 500, 600, 650, 700 and 800 °C. For the samples annealed at 450 °C Raman peaks around 147, 406 and 628 cm^−1^ could be detected. These three peaks should correspond to E_g_, B_1g_, E_g_ of the Raman-active modes of anatase phase. The broad peak around 436 cm^−1^ and the small shoulder around 607 cm^−1^ should correspond to the E_g_ and A_1g_ modes of rutile phase. The above results indicated that the phase transformation between anatase phase and rutile phase could start at 450 °C. With increase of the heat-treatment temperature to 600 °C, the peaks around 443 and 609 cm^−1^ (corresponding to rutile phase) could be also found. This reveals that the anatase phase could gradually transform to rutile phase with increase of the heat-treatment temperature. The rutile modes became stronger while the anatase modes got weaker.

After annealing at 650 °C, a new peak around 244 cm^−1^ appeared distinctly. The Ti-Ni-O demonstrated nearly all of the Raman features of the rutile phase, which indicated that the anatase–rutile transformation was almost completely finished. As the heat-treatment temperature increased to 700 and 800 °C, all of the Raman peaks corresponding to rutile phase became stronger. For the nanotube samples annealed at 800 °C, the titanate peaks around 288 and 706 cm^−1^ were much stronger than those of the samples annealed at 500 °C [23–25].

From the red-shift of the anatase peak (at 147 cm^−1^) in Figure 6, we found that the Raman mode was strongly dependent on the annealing temperature. With increase of the heat-treatment temperature from 450 to 800 °C, the relative intensity of the Raman modes at 147 cm^−1^ decreased and the red-shift of frequency was remarkable. The change for the Raman mode may be attributed to the phase transition. In addition, the gradual relaxation of residual stress during the heat-treatment process could be another factor to cause a frequency red-shift of the Raman mode [26].

The E_g_ and A_1g_ modes (around 447 and 610 cm^−1^, respectively) of the rutile phase were observed when the heat-treatment temperature was gradually changed from 450 to 800 °C. With increase of the heat-treatment temperature the intensity of the E_g_ and A_1g_ modes increased, accompanied by a frequency blue-shift and a decrease of the peak linewidth. As higher heat-treatment temperature could improve the crystalline structure and enhance the degree of crystallinity of the rutile phase, the peak linewidth decreased and the intensity of the E_g_ and A_1g_ modes increased [27]. The blue-shift of the E_g_ and A_1g_ modes of the Ti-Ni-O should be related to a change in the nature of residual stress in the oxide layer [26,28]. In addition, heat-treatment could also result in non-stoichiometry among the components in the Ti-Ni-O oxides. Non-stoichiometry could be one of the important factors to affect lattice vibrational characteristics and cause the frequency blue-shift. Furthermore, phonon confinement effects should be also considered to explain the frequency blue-shift because a nanoscale effect could contribute to frequency blue-shift [29–31].

**Figure 7.**
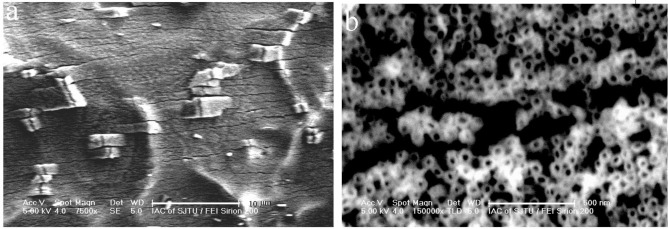
Top view images of the Ti-Ni-O nanotubes after five cycles of bending tests of the nanotube-coated shape memory alloy substrate: (**a**) low magnification image of the microcracks induced in the nanotube array film; (**b**) high magnification image of the crack area.

To investigate the deformation capability of the Ti-Ni-O nanotubes grown on the shape memory alloy, the substrate with as-annealed nanotubes was bent about 80° for five cycles. Figure 7 shows the deformation of the nanotube arrays after the above bending test. As shown in Figure 7a, the Ti-Ni-O film at the most bent area demonstrated a good anti-bending capability by showing partial cracking at local regions and most of the regions did not peel off from the alloy substrate. A closer examination of the cracked areas (Figure 7b) also revealed that the ceramic nanotube arrays could demonstrate a remarkable nanoscale cracking characteristics by showing weak-link cracking behaviors. Obviously, the Ti-Ni-O nanotubes here could have a remarkable deformation ability to withstand severe deformation.

The Ti-Ni-O nanotube arrays grown on the shape memory alloy are expected to find wide applications in both industrial and biomedical occasions. For a real application, mechanical stability of the nanotube arrays has to be considered since a final deformation (such as 5% or 8%) of the shape memory alloy (NiTi substrate) will usually occur during applications. Although the nanotublular oxide film could demonstrate unusual plastic deformation capability due to a nanoscale or nanoporous effect of nanoceramics, deformation behavior of the Ti-Ni-O nanotubes should be further investigated to address such a potential mechanical stability problem. Optimization of the oxide thickness, heat-treatment temperature as well as substrate type (plate or wire) should be realized to minimize a mechanical failure of the nanotube/NiTi alloy system without sacrificing the favorable shape memory effect.

## 3. Experimental Section

### 3.1. Synthesis of Ti-Ni-O Nanotubes

Prior to anodization, all of the Ti-Ni alloy (atomic ratio of Ti and Ni elements was 1:1) samples were ground and polished with 2000# SiC emery papers, and then ultrasonically cleaned with absolute alcohol. Finally, they were rinsed with deionized water and dried in a N_2_ stream. Anodic samples were fabricated with different pulse voltages with a constant frequency of 4000 Hz and duty cycle of 50% for 180 min. We used two kinds of electrolytes, *i.e.*, 5% ethylene glycol/glycerol containing 0.30 M (NH_4_)_2_SO_4_ and 0.4 M NH_4_F, and 5% ethylene glycol/glycerol containing 0.30 M (NH_4_)_2_SO_4_ and 0.2 M NH_4_F. The as-anodized Ti-Ni-O samples were annealed at different temperatures varying from 450 to 800 °C for 1 h to investigate phase transformation of the Ti-Ni-O.

### 3.2. Microstructural Characterization

Surface morphology and composition of the Ti-Ni-O were investigated using a scanning electron microscope (SEM; FEI SIRION 200, FEI Company, Hillsboro, OR, USA) equipped with energy dispersive X-ray analysis (EDXA; OXFORD INCA, Oxford Instruments, Abingdon, Oxfordshire, UK). Phase structures of the as-annealed Ti-Ni-O samples were characterized with a Raman microscope system (Bruker Opties SENTERRA, Bruker Company, Billerica, MA, United States) using an argon ion laser operating at 532 nm.

## 4. Conclusions

In conclusion, highly ordered Ti-Ni-O nanotube arrays were fabricated through pulse anodization of the Ti-Ni shape memory alloy substrates, in a non-aqueous electrolyte of 5% ethylene glycol/glycerol containing 0.30 M (NH_4_)_2_SO_4_ and 0.2 M NH_4_F. The concentration of fluoride in the electrolyte was an important factor for the formation of Ti-Ni-O nanotube arrays. Lower fluoride concentration in the electrolyte was helpful in the formation of Ti-Ni-O nanotube arrays because Ti-Ni-O had a higher corrosion rate for high-concentration electrolyte. This indicated that Ni oxide could easily dissolve in the electrolyte solution. The Raman analysis showed that the Ti-Ni-O annealed at 450 °C, was anatase phase, and the oxide could transform to rutile phase after the annealing treatment at 650 °C. The Ti-Ni-O nanotube arrays had an excellent thermal stability by preserving their nanotubular structures up to 650 °C.

## Figures and Tables

**Table 1.** Composition of the as-annealed Ti-Ni-O nanotubes.

Element	wt%	at%
Ti	46.04	26.26
Ni	14.78	6.83
O	39.18	66.87
